# Type 2 porcine reproductive and respiratory syndrome virus infection increases apoptosis at the maternal-fetal interface in late gestation pregnant gilts

**DOI:** 10.1371/journal.pone.0173360

**Published:** 2017-03-02

**Authors:** Predrag Novakovic, John C. S. Harding, Ahmad N Al-Dissi, Susan E. Detmer

**Affiliations:** 1 Department of Large Animal Clinical Sciences, Western College of Veterinary Medicine, University of Saskatchewan, Saskatoon, Saskatchewan, Canada; 2 Department of Veterinary Pathology, Western College of Veterinary Medicine, University of Saskatchewan, Saskatoon, Saskatchewan, Canada; Xavier Bichat Medical School, INSERM-CNRS - Université Paris Diderot, FRANCE

## Abstract

The pathogenesis of fetal death associated with porcine reproductive and respiratory syndrome (PRRS) is hypothesized to be a consequence of PRRS virus-induced apoptosis at the maternal-fetal interface (MFI). The objectives of this study were to evaluate distribution and degree of apoptosis in the uterine and fetal placental tissues during the experimental type 2 PRRS virus (PRRSV) infection and determine associations between apoptosis at the MFI, PRRSV RNA concentration and antigen staining intensity, PRRSV-induced microscopic lesions, and fetal preservation status. A total of 114 naïve, high-health pregnant gilts were inoculated with type 2 PRRSV on gestation day 85±1 with euthanasia 21 days later; 19 sham-inoculated gilts served as controls. Two hundred and fifty samples of uterine tissue with fetal placenta were selected based on negative, low PRRSV RNA, and high PRRSV RNA concentration (0, < or > 2.7 log_10_ copies/mg, respectively). TUNEL assay was used to detect apoptosis in the endometrium and at the MFI. PRRSV RNA concentration and numbers of PRRSV immunopositive cells in uterine and placental tissue were positively associated with the severity of apoptosis in the endometrium and the MFI (P<0.001, P<0.05 and P<0.001, respectively). The number of TUNEL positive cells at the MFI was also positively associated with the severity (P<0.001) of vasculitis, but not total numbers of inflammatory cells in the endometrium. Increased numbers of TUNEL positive cells at the MFI were associated with PRRSV load in the fetal thymus, and greater odds of meconium staining of the fetus at 21 days post infection (P<0.001 for both). These findings suggest an important role of apoptosis in the pathogenesis of uterine epithelial and trophoblastic cell death at the MFI. Moreover, apoptosis at the MFI is significantly associated with fetal demise during *in utero* type 2 PRRSV infection.

## Introduction

Despite nearly 25 years of research work, the underlying mechanism of fetal death in the reproductive form of porcine reproductive and respiratory syndrome (PRRS) is still undetermined. Recent studies begin to challenge the previous understanding of fetal death as a result of PRRS virus (PRRSV) replication in the fetal tissues, proposing conversely that pathological processes at the maternal-fetal interface (MFI) could play a role in fetal death [1, 2].

Apoptosis is a well-described mechanism of cell death in the pathogenesis of viral infections [3]. Viruses induce apoptosis of susceptible cells to promote viral spread in the tissue and prevent efficient immune response [3]. Induction of pathological apoptosis in human fetal membranes has been confirmed to be associated with reproductive failure [4].

PRRSV infection has been reported to cause apoptosis within cell lines *in vitro* [5]. Additionally, several *in vivo* studies revealed the presence of apoptotic cells widely distributed in tissues such as lungs, testes, lymph nodes, and thymus during postnatal PRRSV infection of nursing and growing pigs [6–8]. More specifically, it has been observed that majority of cells undergoing apoptosis during the PRRSV infection are macrophages and lymphocytes [6]. However, the number of apoptotic cells detected in the lymphatic tissue of experimentally PRRSV-infected growing pigs was reported to be higher than a number of infected cells, indicating that PRRSV can induce apoptosis not only directly in susceptible subpopulation of local tissue macrophages [9, 10], but also indirectly in non-permissive cells by some yet unknown mechanism [11].

The role of apoptosis in the pathogenesis of reproductive PRRS has been rarely investigated. So far, only one study of type 1 PRRSV infection of pregnant sows confirmed increased apoptosis in infected macrophages and surrounding cells in the fetal placental tissue and endometrium at the maternal-fetal interface [1]. However, the question of the role of PRRSV-induced apoptosis in the fetal implantation sites in the pathogenesis of the fetal death still remains unanswered. More specifically, the study of the relationship between the extent of apoptosis at the maternal-fetal interface and PRRS virus concentration in different reproductive compartments, along with fetal preservation status needs to be conducted in order to further elucidate the importance of apoptosis in PRRSV-induced reproductive failure. Even more, the related experimental study of type 2 reproductive PRRSV infection is needed due to significant differences in pathogenicity between type 1 and type 2 PRRS virus genotypes. [12]

Results from our recent experimental study of reproductive type 2 PRRSV infection of late gestation pregnant gilts at 21 days post-infection (DPI) confirmed that severe pathological lesions in the uterus with fetal placenta consisting of lymphohistiocytic endometritis, vasculitis and placental detachments were more frequent than any fetal lesions [13]. In order to investigate the potential role of apoptosis in the pathogenesis of PRRSV-induced reproductive failure, we have developed three objectives for the present study. The first objective of the present study was to evaluate the types and numbers of cells undergoing apoptosis in the endometrium and maternal-fetal interdigitation areas, and to assess the differences between groups of uterine tissue samples selected on the basis of type 2 PRRSV viral load (negative, low, high). The second objective was to evaluate the potential relationships between the numbers of apoptotic cells at MFI and severity of the inflammation affecting endometrium and related vasculature. Finally, the third objective examined the relationship between the apoptosis at MFI, PRRSV viral load in the fetal thymus and the preservation status of the corresponding fetus.

## Material and methods

### Ethics statement

Inoculation of gilts or sows in the last trimester of gestation is a widely accepted and commonly used model for studying reproductive PRRS and currently there are no available alternative models [1, 2, 14–17]. Fetal stress and discomfort *in utero* are presently not possible to monitor in the litter bearing species. However, the fact that fetal death can occurr at any time during the experimental infection was carefully considered in the preparation for this study by planning the minimal gilt numbers which can allow both deep phenotyping and genotyping of gilts and fetuses. Humane intervention point (HIP) checklist was also developed for this project which included clinical monitoring of pregnant gilts on daily basis [13]. Protocol for live animal trial of this experiment was fully designed in accordance with Canadian Council on Animal Care guidelines for humane animal use and it was approved by the University of Saskatchewan’s Animal Research Ethics Board (permit #20110102). Gilts used in this study were purebred Landrace gilts from a high-health nucleus herd which was confirmed clinically and serologically to be free of PRRSV, *Mycoplasma hyopneumoniae* and *Actinobacillus pleuropneumoniae*. Additionally, all gilts were vaccinated against porcine parvovirus, erysipelas twice before the breeding, porcine circovirus type 2 at 3 weeks of age, and *Haemophilus parasuis* at 9 and 22 weeks of age [18].

### Experimental design

The experimental protocol for this study has been previously described in detail [18]. Briefly, on 85±1 gestation day, 114 PRRSV-naïve pregnant gilts were intramuscularly and intranasally inoculated with PRRSV (10^5^ TCID50 total dose, NVSL 97–7895 [18], GenBank Accession No. AF325691) and 19 negative control pregnant gilts were sham inoculated with minimum essential medium [19]. At 21 DPI, dams and their litters were humanely euthanized for necropsy examination. Gilts were humanely euthanized by intravenous barbiturate overdose (30 mL Euthanyl Forte supplying 16,200 mg pentobarbital sodium per gilt; Vetoquinol, Lavaltrie, QC) followed by cranial captive bolt, pithing and exsanguination. As pentobarbital readily crosses the placenta and provides concentrations similar to that in maternal serum, fetuses were also euthanized by barbiturate overdose within ~2–3 minutes of administration. The reproductive tract was removed intact and cut opened from the tip of the horns. Each fetus was removed along with its umbilical cord, placenta and a portion of the uterus adjacent to the umbilical stump.

### Assessment of fetal preservation status

The preservation status of each live fetus was assessed as previously described [18] based on the external gross appearance of its skin and umbilical cord as: viable (VIA; normal, white to purple skin with visible hair and regular umbilical cord), and meconium-stained (MEC; skin covered with inspissated, brownish amniotic fluid and regular umbilical cord with edema) [13]. All dead fetuses (partially decomposed to autolyzed) were excluded from this study since the MFI was compromised.

### RT-qPCR quantification of PRRSV RNA in the uterine-placental tissue and fetal thymus

Samples of fetal thymus and uterine-placental tissue (uterine endometrium with adherent placental layers) collected 4 cm from the umbilical attachment of each live fetus were analyzed by strain-specific in-house RT-qPCR to determine PRRSV RNA concentration as previously described [18]. Specific primers for a conserved region of ORF7 of strain NVSL 97–7895 were designed. Results were reported as logarithm base 10 target RNA concentration per mg of tissue.

### Selection of uterine-placental tissue samples

From 679 available uterine-placental tissue samples which met the criteria of size (≥ 1 cm) and fully attached fetal placenta, a subset of 250 was selected based on the PRRSV RNA concentration measured in the uterine-placental tissue for TUNEL assay and microscopic assessment of severity of inflammation. Three groups were formed: negative (50 samples from PRRSV non-infected pregnant gilts, PRRSV RNA not detected), low (100 samples from PRRSV-infected pregnant gilts, PRRSV RNA concentration less than 2.7 log10 copies per mg), and high (100 samples from PRRSV-infected pregnant gilts, PRRSV RNA concentration greater than 2.7 log10 copies per mg). The low and high viral samples of uterine-placental tissue corresponding to the individual fetus were matched pairs from 61 PRRSV-infected gilts; one pair from 24 gilts and two pairs from 38 gilts. Fifty uterine-placental samples corresponding to different fetuses were selected from six negative control gilts (9 uterine-placental samples from 5 gilts, 5 uterine-placental samples from 1 gilt).

### TUNEL assay of uterine-placental tissue

The detection of DNA fragmentation due to apoptosis was performed on all 250 tissue sections using Terminal deoxynucleotidyl transferase-mediated dUTP nick end labeling (TUNEL) assay as previously described at room temperature [20]. Briefly, after deparaffinization and rehydration of 5 μm microsections of formalin-fixed paraffin embedded (FFPE) MFI tissue, protein digestion was done using Proteinase K solution (Dako, Carpinteria, CA) for 15 min. Endogenous peroxidase activity was blocked using 3% hydrogen peroxide for 5 min. Equilibration buffer provided with the ApopTag Plus Peroxidase In Situ Apoptosis Detection Kit (Millipore, Etobicoke, Ontario) was immediately applied for 10 sec at room temperature, followed by terminal deoxynucleotidyl transferase (TdT) enzyme at 37°C for 1 hour in a humidity chamber. The slides were washed with stop/wash buffer for 15 sec and incubated for 10 min. After that, they were washed three times with phosphate buffered saline with tween20 (Fisher Scientific, Markham, Ontario) for 1 min and incubated with the anti-digoxigenin conjugate for 30 min. The signal was revealed using 3-Amino-9-Ethylcarbazole (AEC) chromogen (Dako) for 15 min and sections were counterstained with Mayer’s hematoxylin (Fisher Scientific) and coverslipped.

### PRRSV immunohistochemistry

Immunohistochemistry (IHC) for detection of PRRSV antigen was performed on all 250 tissue sections as previously described [19] using mouse monoclonal antibody against PRRSV nucleocapsid N protein at dilution 1:200 (SDOW17, Rural Technologies Inc., Brookings, USA).

### Microscopic assessment of inflammation in uterine-placental tissue

Assessment of the inflammation within the lamina propria of the endometrium of the uterus was performed by using a grid and randomly selecting three 200x magnification fields. Within each of these fields, numbers of inflammatory cells were counted at 600x magnification from three 3x3 boxes (one 3x3 box area size is equivalent to 0.05 mm^2^). Total numbers of inflammatory cells were obtained by adding all counted inflammatory cells from the total of nine 3x3 boxes in the grid.

Inflammation of the vasculature in the uterus was assessed based on two criteria. Distribution of vasculitis was assessed in each of the three randomly selected 200x magnification fields by counting the total number of vessels (usually between 3 and 15) larger than smallest box within the 10x10mm grid (one box in the grid equals 0.05 mm^2^ area size, while the whole grid is 0.5 mm^2^ area size). Then, the number of vessels affected by vasculitis were counted and divided by the total number of blood vessels. Scores for severity of vasculitis for the selected samples were previously published [13]. The score was based on the presence of inflammatory cells in the blood vessel wall with concomitant vacuolar degeneration and/or necrosis of the cells in the blood vessel wall layers (intima, media, and adventitia) as previously described [13]. Briefly, in the randomly selected three 200X microscopic fields of endometrium per uterine tissue section three blood vessels were scored at 400X magnification as: grade 1 = only mild inflammation in wall; grade 2 = moderate inflammation in wall with vacuolation and splitting of smooth muscles or necrosis of wall layers; grade 3 = severe inflammation in wall with degeneration and necrosis in the blood vessel wall layers.

### Image analysis

Quantitative analyses of TUNEL and PRRSV antigen IHC staining was performed using an Image-Pro Plus, version 7 software (Media Cybernetics, Inc., Rockville, MD, USA) as previously described [19]. Briefly, ten microscopic fields of the endometrium, captured using a 20X microscope objective lens. The randomly selected areas represented 1 mm^2^ area (10 mm^2^ total/slide). Multiple polygonal fields (total area size of 3–4 mm^2^) were randomly selected at the MFI (uterine and fetal placenta interdigitation area). Inside those selected fields, total numbers of TUNEL positive cells were manually counted and averaged per 1 mm^2^ area size for the statistical analyses.

### Statistical analysis

Separate two-level, linear mixed-effects regression models were developed controlling for litter of origin (gilt identification) as a random effect using Stata v13 (StataCorp LP, TX, USA) to These models were used to analyze if numbers of TUNEL positive cells in the endometrium and MFI differed among PRRS viral load groups (negative, low, high). Additionally models evaluated the associations between the TUNEL positive cells in the endometrium and MFI and numbers of PRRSV immunopositive cells in the PRRSV-infected endometrial and fetal placental samples. For these models, TUNEL positive cells in the endometrium and MFI, and PRRSV immunopositive cells in the endometrium and fetal placenta were zero-skewness log (lnskew0) transformed to ensure that model assumptions of linearity and homogeneity were not violated. A similar model was used to assess relationships between TUNEL positive cells in the MFI and PRRS viral load in fetal thymus. In this model, the outcome variable (number of TUNEL positive cells at MFI) was natural logarithm transformed and fetuses from negative control gilts were excluded.

The relationship between numbers of TUNEL positive cells determined at the MFI of PRRSV-infected samples (low and high viral load groups) and endometrial inflammation, vasculitis severity and distribution, and PRRS viral load in uterine-placental tissue was also assessed using two-level, linear mixed-effects regression models. Firstly, the four non-correlated, biologically plausible potential predictor variables: severity and distribution of vasculitis, total numbers of inflammatory cells in the endometrium and PRRS viral load in uterine-placental tissue, were confirmed to be unconditionally associated with the number of TUNEL positive cells (P<0.001 for all). All four variables were subsequently placed in a full model, and a backward, stepwise elimination performed to a parsimonious final model. In all models, the outcome variable (number of TUNEL positive cells) was natural logarithm transformed to avoid violation of the normality and homogeneity model assumptions.

Finally, generalized estimating equations (GEE) accounting for gilt of origin (family-binomial, link-logit, correlation-exchangeable) were used to determine if the TUNEL positive cell count at the MFI was related to the odds of a fetus being MEC (versus VIA). Firstly, a model including 200 PRRSV-infected fetuses (26 MEC, 174 VIA) was run. Given the small number of MEC (n = 26) relative to VIA (n = 174) fetuses in this initial model, a series of smaller, balanced GEE models were then developed in which the 26 MEC were matched with 26 randomly selected VIA fetuses. Six balanced GEEs were run, each using a different set of 26 randomly selected VIA fetuses; 156 fetuses in total or 90% of the VIA population. The coefficients, SE, P values were compared across all models to ensure consistency and are reported herein.

## Results

### Distribution of TUNEL staining in the uterine-placental tissue

**Fig 1:**
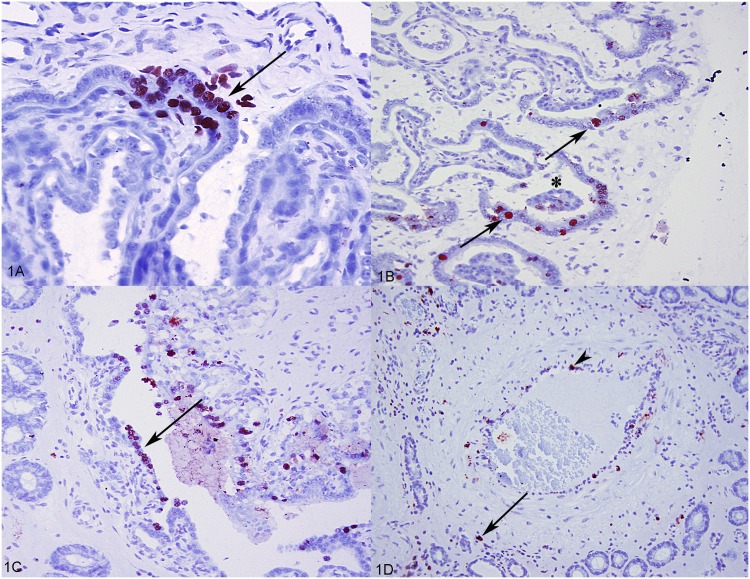
TUNEL assay in the uterine-fetal placental tissues from PRRSV-infected pregnant gilts. (A) Focal area of apoptosis (arrow) affecting both the trophoblast and uterine epithelial cells at the uterus-fetal placenta interface from PRRSV-infected pregnant gilt. (B) Multiple apoptotic cells (arrows) closely associated with the area of microseparation between chorioallantois and uterus (asterisk) from PRRSV-infected gilt. (C) Apoptotic uterine luminal epithelial and trophoblastic cells in the area of severe separation of the chorioallantois from the uterus (arrow). (D) Single cell apoptosis of inflammatory cells in the endometrium (arrow); endometrial artery with the apoptotic endothelial cell (arrowhead).

TUNEL positive (apoptotic) cells were variably distributed throughout the uterine tissue with the fetal placenta, but primarily in the endometrial-placental junctional areas. TUNEL positive cells at the maternal-fetal interface ranged from focal areas involving single cell apoptosis of trophoblast on the fetal side of the placenta, and uterine epithelium on the maternal side (Fig 1A) to multifocal areas of multiple cell apoptosis that were clearly associated with microseparations (Fig 1B). In rare instances, more severe separation of the placenta from the uterine epithelium was evident with increased numbers of apoptotic cells of uterine epithelium and fetal trophoblast (Fig 1C). Single cell apoptosis was also randomly distributed in the endometrium, affecting mostly mild to moderate numbers of inflammatory cells; predominantly lymphocytes and macrophages (Fig 1D). Occasionally individual cells of the uterine glandular epithelium were affected as well. Rare endothelial cells of the inflamed blood vessels demonstrated strong TUNEL positivity (Fig 1D).

**Fig 2:**
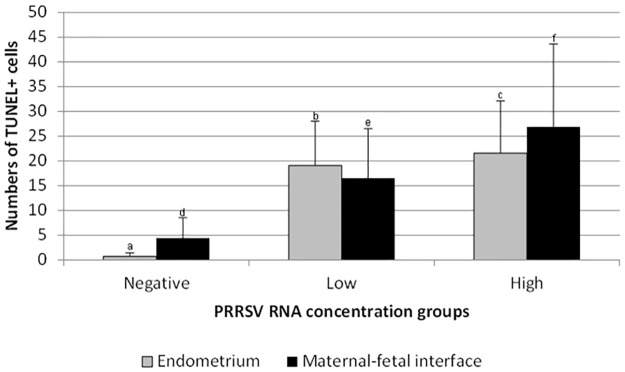
Mean numbers of TUNEL (apoptotic) positive cells per 1 mm^2^ of the endometrium and maternal-fetal interface. Superscript letters (^a, b, c^ or ^d, e, f^) indicate significant differences (*P* < 0.05) between PRRSV viral load groups. Error bars represent standard deviation.

Numbers of TUNEL positive (apoptotic) cells in the endometrium and MFI were both positively associated with PRRS viral load groups (*P*<0.001, for both). The number of TUNEL positive (apoptotic) cells was greater in the high viral load compared to low viral load group, in both the endometrium and MFI. While also detected in the uterine tissue samples of negative control gilts, TUNEL cell counts were markedly lower in both endometrium and MFI (Fig 2) compared to infected gilts. Similar results were found with PRRSV antigen staining. The numbers of TUNEL positive cells in the endometrium was positively associated with the numbers of PRRSV immunopositive cells in the endometrium (*P*<0.001), while numbers of TUNEL positive cells at the MFI was positively associated with both the numbers of PRRSV immunopositive cells in the endometrium and fetal placenta (*P*<0.05, and *P*<0.001, respectively).

### Relationship of TUNEL positive cell counts at MFI to microscopic lesions and PRRSV load in uterine-placental tissue

The most frequent and consistent microscopic finding was lymphohistiocytic endometritis which was characterized by the presence of large numbers of lymphocytes mixed with moderate numbers of histiocytes and lesser numbers of plasma cells infiltrating lamina propria (stroma), uterine glands, uterine epithelium interdigitations and endometrial blood vessels [13]. No microscopic evidences of neutrophilic (purulent) inflammation nor presence of bacterial organisms have been observed in the uterine and fetal placental samples obtained both from negative controls and PRRSV-infected pregnant gilts.

The variables unconditionally associated with the number of TUNEL positive cells at the MFI included: vasculitis distribution and severity score, the total number of inflammatory cells in the endometrium and PRRS viral load in the uterine-placental tissue. Of these, only severity of vasculitis in the uterine tissue and PRRS viral load in the uterine-placental tissue were confirmed to be significantly positively associated with the number of TUNEL positive cells at MFI.

### Relationship of TUNEL positive cell counts at MFI to PRRSV load in the fetal thymus and fetal preservation status

Numbers of TUNEL positive cells at the MFI were positively associated with PRRSV RNA concentration in fetal thymus with (*P*<0.001). For each 1 log_10_ increase in PRRSV RNA concentration (copies/mg) in fetal thymus, the number of TUNEL positive (apoptotic) cells increased 1.089 (e^0.085^) cells/mm^2^ of MFI.

Regarding the relationship between TUNEL positive cells count at the MFI and fetal preservation status, all GEE models result were consistent in confirming that an increase in TUNEL positive cell count at the MFI significantly increased the odds of a fetus being meconium stained compared to being viable (S1 Table). In the inclusive model of 200 PRRSV-infected fetuses (26 MEC, 174 VIA), each addition TUNEL positive cell per 1 mm^2^ at the MFI increased the odds of a fetus being meconium stained by 1.08 times (95% CI 1.05–1.12). A similarly and consistent increase in the odd ratio of meconium staining was noted across all of the smaller balanced models that included 26 meconium stained and 26 viable fetuses (OR = 1.06 to 1.15 depending on the model; S1 Table).

## Discussion

A better understanding of the pathological processes at the maternal-fetal interface during type 2 PRRSV infection is needed to understand the pathogenesis of PRRSV-induced fetal death. In a related study, we have confirmed that type 2 PRRSV infection causes significant microscopic lesions in the uterus and fetal placenta [13]. The goal of this study was to evaluate the distribution and extent of apoptosis in the uterine and fetal placental tissues during type 2 PRRSV infection, evaluate the potential associations between the numbers of apoptotic cells in the endometrium and maternal-fetal interface and PRRSV viral load in uterine-placental tissue and fetal thymus, microscopic lesions, and fetal preservation status.

The TUNEL analyses undertaken in the present study confirmed that the numbers of apoptotic cells in uterine tissue and fetal placenta significantly increased with PRRS viral load in the uterine-placental tissue. Although the apoptosis was a very prominent feature in uterine and fetal placental tissue, it appears that it affects morphologically different cell types. In the *lamina propria* of the endometrium, the majority of the cells undergoing apoptosis were inflammatory cells, macrophages and lymphocytes. Since lymphocytes have not been reported as being PRRSV-permissive, and our related study confirmed that low numbers of PRRSV antigen-positive macrophages in the *lamina propria* [19], it is possible that significant apoptosis of individual lymphocytes may be the indirect effect of PRRSV replication in the susceptible macrophages [21] possibly caused by secretion of pro-apoptotic cytokines, e.g. TNFα [22]. On the other hand, the largest numbers of apoptotic cells at maternal-fetal junction comprised of fetal trophoblastic epithelial cells followed by uterine epithelial cells. This finding, consistently present throughout all uterine/fetal placental samples obtained from PRRSV infected gilts, and rare in negative control gilts, is in agreement with previously published results for type 1 PRRSV infection [1]. Also, this pattern of distribution of apoptosis at maternal-fetal interface is highly similar to the pattern of distribution of cells demonstrating strongest immunopositivity for PRRS viral nucleoprotein detected in our recently published study of PRRSV routes of transplacental transmission [19]. Increased single-cell apoptosis of trophoblastic and uterine epithelial cells at MFI can have a detrimental effect on the epithelium integrity through the loss of cell to cell and cell to extracellular matrix adhesion, and may potentially initiate more pronounced physiological apoptosis (anoikis; responsible for normal tissue reorganization) of neighboring cells [23]. Both anoikis and pathological PRRSV-induced apoptosis could perpetuate cellular loss at the maternal-fetal interface, and accelerate the placental detachment from the uterus. Additionally, cellular death in these areas could initiate accentuated inflammatory cell reaction and release of pro-inflammatory and pro-apoptotic cytokines that in turn could further exacerbate fetal placental detachment. That being said, marked apoptosis was frequently observed closely associated with areas of severe detachment of the chorioallantois from uterine epithelium, which could indicate a mechanism of placental separation.

Apoptosis of uterine epithelial and fetal trophoblastic cells of the interface was significantly associated with PRRS viral load in the uterine-placental tissue and fetal thymus; a key finding of this research. Furthermore, this study has confirmed a a similar positive association between numbers of apoptotic cells at the maternal-fetal interface detected by TUNEL assay, and numbers of PRRSV antigen immunopositive cells in uterine endometrium and fetal placenta detected by immunohistochemistry. The fact that cell death at MFI is associated with PRRSV concentration in the fetus is novel and suggests complex relationship among PRRS infection of multiple reproductive compartments and apoptosis at MFI may exist. Nevertheless, increased apoptosis of uterine epithelial and fetal trophoblastic cells of fetal placenta could potentially compromise the maternal-fetal barrier causing subsequent detachment of the fetal placenta from the uterus, which along with other factors such as PRRSV infection of the fetus [24] and fetal lesions and umbilical lesions [13] could represent additional factors contributing to fetal demise during *in utero* type 2 PRRSV infection. In accordance with this finding, our study also confirmed a significant, positive relationship between the degree of apoptosis at the MFI and meconium staining of the fetus, an early pathologic indicator of fetal compromise [25]. More specifically, higher numbers of apoptotic cells at the MFI increased the odds of a fetus being meconium stained compared to viable.

Our study also confirmed a positive association between the severity of type 2 PRRSV-induced vasculitis and apoptosis at the MFI. When the total numbers of inflammatory cells in the lamina propria of the endometrium, distribution of vasculitis and severity score of vasculitis in the uterine tissue samples were offered in the statistical model used to predict numbers of apoptotic cells at the MFI, only severity of vasculitis was positively related to the numbers of apoptotic cells. These results indicate a potential role of PRRSV-induced vasculitis and associated damage to blood vessels, rather than severity of inflammation in the endometrium, in the pathogenesis of the cell death at MFI. Furthermore, this suggests that hematotroph, which plays an essential role in the development and growth of the porcine fetus, may be compromised during PRRSV *in utero* infection. This may be one reason why fetal compromise associated with PRRSV infection appears to disproportionately affect large fetuses rather than small fetuses, and why the body weight of surviving fetuses is decreased following transplacental PRRSV infection [26]. Vascular dysfunctions causing hypoperfusion and ischemia have already been implicated in human pregnancies complicated by preeclampsia, placental abruption, and fetal growth restriction [27]. Whether or not PRRS virus-induced vascular inflammation could impair blood perfusion to such degree to lead to severe metabolic derangements of uterine and trophoblastic epithelial cells of the porcine epitheliochorial placenta requires further investigation. The results of the present study, however, suggest that in addition to PRRSV-mediated apoptosis at the MFI, PRRSV-induced vascular lesions may be a supplementary pathogenic mechanism that exacerbates damage to the maternal-fetal barrier.

In summary, this study of type 2 PRRSV infection of third-trimester pregnant gilts confirmed a significant increase in the numbers of apoptotic cells in PRRSV-infected uterine and fetal placental tissue. This study also determined that increased numbers of apoptotic cells at the maternal-fetal interface are significantly associated with PRRS RNA concentration in the fetus, and odds of fetal meconium staining. Also, a significant positive association between the apoptosis at the maternal-fetal interface and the severity of PRRSV-induced vasculitis in the endometrium was confirmed. This research helps to advance the understanding of the pathogenesis of fetal demise in type 2 reproductive PRRS.

## Supporting information

S1 Table Results of Generalized Estimating Equations (GEE). GEE confirmed the association between numbers of TUNEL positive cells/mm^2^ at maternal fetal interface (MFI) and the odds of a meconium stained fetus (versus viable).
(DOCX)
