# Supplementary material for: Type 2 porcine reproductive and respiratory syndrome virus infection increases apoptosis at the maternal-fetal interface in late gestation pregnant gilts
Source: PLoS One. 2017 Mar 2;12(3):e0173360. doi: 10.1371/journal.pone.0173360 (PMC5333878; doi:10.1371/journal.pone.0173360)
Supplement: S1 Table — GEE confirmed the association between numbers of TUNEL positive cells/mm2 at maternal fetal interface (MFI) and the odds of a meconium stained fetus (versus viable). (DOCX) [file pone.0173360.s001.docx]

**S1 Table.** Results of Generalized estimating equations (GEE) confirming the association between numbers of TUNEL positive cells/mm^2^ at maternal fetal interface (MFI) and the odds of a meconium stained fetus (versus viable).

| **GEE model*** | **No.^a^** | **Coefficient** | **Odds Ratio** | **SE** | ***P* values^†^** |  | **95% CI** |  |
| --- | --- | --- | --- | --- | --- | --- | --- | --- |
| **Inclusive** | 200 | 0.08 | 1.08 | 0.015 | <0.001 |  | 0.051, 0.109 |  |
| 1 | 52 | 0.074 | 1.08 | 0.021 | <0.001 |  | 0.033, 0.115 |  |
| 2 | 52 | 0.136 | 1.15 | 0.04 | <0.001 |  | 0.058, 0.214 |  |
| 3 | 52 | 0.093 | 1.10 | 0.026 | <0.001 |  | 0.042, 0.143 |  |
| 4 | 52 | 0.066 | 1.07 | 0.021 | 0.001 |  | 0.256, 0.106 |  |
| 5 | 52 | 0.062 | 1.06 | 0.02 | 0.002 |  | 0.023, 0.101 |  |
| 6 | 52 | 0.101 | 1.11 | 0.029 | 0.001 |  | 0.044, 0.159 | |
| **AVG of 6 balanced GEE models** |  | 0.089 | 1.09 |  |  |  |  | |

^*^ A model with 200 fetuses ("Inclusive") including 26 meconium stained (MEC) and 174 viable (VIA) was initially run followed by six balanced GEE models including 26 MEC fetuses and a different set of 26 randomly selected VIA fetuses.

^†^ Statistical significance was assigned at the *P* < 0.05 level.

^a^ Total number of fetuses analyzed in the model
